# A recessive variant of *XRCC4* predisposes to non-*BRCA1/2* breast cancer in chinese women and impairs the DNA damage response via dysregulated nuclear localization

**DOI:** 10.18632/oncotarget.2623

**Published:** 2014-10-22

**Authors:** Min He, Xin Hu, Li Chen, A-Yong Cao, Ke-Da Yu, Ting-Yan Shi, Xia-Ying Kuang, Wen-Biao Shi, Hong Ling, Shan Li, Feng Qiao, Ling Yao, Qingyi Wei, Gen-Hong Di, Zhi-Ming Shao

**Affiliations:** ^1^ Department of Breast Surgery, Key Laboratory of Breast Cancer in Shanghai, Fudan University Shanghai Cancer Center, Shanghai, China; ^2^ Department of Gynecologic Oncology, Fudan University Shanghai Cancer Center, Shanghai, China; ^3^ Cancer Institute, Fudan University Shanghai Cancer Center, Shanghai, China; ^4^ Department of Oncology, Shanghai Medical College, Fudan University, Shanghai, China; ^5^ Duke Cancer Institute, Duke University Medical Center, Durham, NC, United States of America

**Keywords:** XRCC4, homozygous variant, nuclear localization, susceptibility, breast cancer

## Abstract

*XRCC4* plays a crucial role in the non-homologous end joining pathway that maintains genome stability. In this two-stage case-control study with 1,764 non-*BRCA1/2* breast cancer patients and 1,623 cancer-free controls, we investigated the contribution of genetic variants of *XRCC4* to breast cancer susceptibility in Chinese women. We identified a recessive missense variant, rs3734091 (c.739G>T, p.Ala247Ser), of *XRCC4* that was significantly associated with an increased risk of breast cancer (odds ratio [OR] = 3.92, *P* = 0.007), particularly with the risk of developing triple-negative breast cancer (OR = 18.65, *P* < 0.0001). This p.Ala247Ser variant disturbed the nuclear localization of XRCC4 in cells homozygous for the rs3734091-T allele but not in heterozygous cells at both the cellular and tissue levels. In heterozygous cells, wild-type XRCC4 facilitated the nuclear localization of the XRCC4^A247S^ mutant, thus compensating for the impaired localization of XRCC4^A247S^. This provided a biological mechanism by which rs3734091 conferred an increased susceptibility to non-*BRCA1/2* breast cancer exclusively under a recessive model. Further functional analyses revealed that p.Ala247Ser impaired the DNA damage repair capacity and ultimately perturbed genomic stability. Taken together, our findings document the role of *XRCC4* in non-*BRCA1/2* breast cancer predisposition and reveal its underlying biological mechanism of action.

## INTRODUCTION

Breast cancer is the most common malignancy in women worldwide. Genetic mutations in *BRCA1* and *BRCA2* account for approximately 20% of breast cancer cases. Although mutations in additional genes (such as *PALB2*, *CHEK2*, and *ATM*) and novel loci identified through genome-wide association studies (GWAS) have also been found to predispose people to breast cancer, together these alleles only confer a minor increase in risk in non-*BRCA1/2* families [1-3]. Therefore, it is important to identify the cancer predisposition factors in non-*BRCA1/2* breast cancer.

Most breast cancer susceptibility genes are involved in the maintenance of genomic integrity and double-strand break (DSB) repair [3]. In eukaryotic cells, non-homologous end-joining (NHEJ) and homologous recombination (HR) constitute the two major mechanisms for DSB repair [4]. There has been increasing interest in the role of DSB repair genes in both cancer susceptibility and tumor development. In our previous studies, we screened a series of breast cancer susceptibility genes within the HR repair pathway, including *BRCA1/2* [5], *PALB2* [6], *RAD50*, and *NBS1* [7], in Chinese women. However, the genetic deficiencies in these genes accounted for no more than 10% of the genetic basis for breast cancer in Chinese women, indicating the presence of genetic heterogeneity in the susceptibility genes in different racial/ethnic populations. Therefore, it is likely that focusing on the NHEJ repair pathway may lead to the identification of additional susceptibility loci related to non-*BRCA1/2* breast cancer.

The multistep process of NHEJ involves a well-defined set of proteins, including the Ku70/80 heterodimer, the DNA-dependent protein kinase catalytic subunit (DNA-PKcs), and the XRCC4-Ligase IV-XLF complex, which functions as a key catalyzer of the final ligation step in the NHEJ pathway [8, 9]. XRCC4 tightly associates with Ligase IV to both stabilize Ligase IV from degradation and to stimulate its adenylation of Ligase IV [10-12]. XLF is a novel NHEJ factor that participates in the XRCC4-Ligase IV complex via a direct interaction with XRCC4 [13, 14]. Furthermore, XRCC4 exhibits intrinsic DNA-binding activity [12] and interacts with polynucleotide kinase [15]. Disrupting *XRCC4* in mouse embryonic cells results in reduced proliferation and radiation hypersensitivity but may also give rise to chromosomal instability [16]. Thus, XRCC4 is a multifaceted protein with pivotal roles in NHEJ repair signaling and genomic integrity.

Large studies on the role of XRCC4 single-nucleotide polymorphisms (SNPs) in cancer susceptibility have been performed in hepatocellular carcinoma [17], lung cancer [18], multiple myeloma [19], and oral cancer [20]. Notably, there have only been a few studies on the associations between genetic variants of *XRCC4* and breast cancer susceptibility; these studies were performed regardless of the *BRCA1/2* status, and the results were inconclusive [21-23]. Furthermore, the biological underpinnings of these genetic associations have not yet been well established.

In this study, we aimed to comprehensively evaluate the associations between *XRCC4* genetic variants and non-*BRCA1/2* breast cancer risk in a two-stage case-control study. We identified a missense variant (c.739G>T, p.Ala247Ser) of *XRCC4* that correlated with an increased risk of non-*BRCA1/2* breast cancer. We also examined the underlying biological mechanism of action by which this variant caused a pathogenic alteration in the DNA repair response exclusively under a recessive model. The current study identified *XRCC4* as a non-*BRCA1/2* breast cancer susceptibility gene in the Chinese population.

## RESULTS

### Associations between *XRCC4* variants and the risk of non-*BRCA1/2* breast cancer

In the first discovery cohort, we genotyped four potential functional SNPs (rs3734091, rs56334522, rs28360342, and rs2035990) in 562 non-*BRCA1/2* breast cancer patients and 504 controls. The criteria used to select these SNPs are described in the Materials and Methods section. All the observed genotype distributions among the controls were in Hardy-Weinberg equilibrium (HWE). Although none of the genotyped SNPs exhibited a significant difference in allele frequency between the cases and controls (Supplemental Table 1), the genotype distributions revealed that rs3734091 was significantly associated with breast cancer under a recessive model (Supplemental Table 2). Compared with the common homozygote CC, the rs3734091-TT genotype correlated with an increased risk of breast cancer.

To confirm this finding, we subsequently performed a validation study in an independent cohort of 1,202 cases and 1,119 controls. Under a recessive model, the rs3734091-TT genotype showed a consistent association with an increased risk of breast cancer (odds ratio [OR] = 3.07, 95% CI 0.98-9.59, *P* = 0.038; Table 1). This association became more significant after the two sets were combined (OR = 3.92, 95% CI 1.30-11.83, *P* = 0.007), with an adequate statistical power of 92%.

**Table 1:** Association of rs3734091 with breast cancer risk in the discovery and validation studies

Study	Genotype	Controls (%)	Cases (%)	Dominant model		Recessive model	
				OR^a^ (95% CI)	*P*^a^	OR^a^ (95% CI)	*P*^a^
Discovery study							
	GG	435 (87.3%)	475 (87.5%)	1.00 (0.69-1.45)	0.99	NA (0.00-NA)	**0.019**
	GT	63 (12.7%)	64 (11.8%)				
	TT	0 (0%)	4 (0.7%)				
Validation study							
	GG	1,000 (91%)	1,049 (89%)	1.31 (0.99-1.73)	0.058	3.07 (0.98-9.59)	**0.038**
	GT	95 (8.6%)	117 (9.9%)				
	TT	4 (0.4%)	12 (1%)				
All cases (Discovery + Validation)							
	GG	1,421 (89.8%)	1,503 (88.5%)	1.14 (0.91-1.42)	0.25	3.92 (1.30-11.83)	**0.007**
	GT	158 (10%)	180 (10.6%)				
	TT	4 (0.2%)	16 (0.9%)				

NOTE: Bold values denote *P* ≤ 0.05.

a The *P* value, OR, and 95% CI were calculated using logistic regression and were adjusted for age, age at menarche, age at primiparity, menopause status, family history of breast cancer, and BMI.

Abbreviations: NA, not applicable.

### The rs3734091-TT genotype confers a high risk for triple-negative breast cancer (TNBC)

Stratifying the breast tumors by ER status indicated that the rs3734091-TT genotype was strongly associated with ER-negative breast cancer (OR = 9.43, 95% CI 2.91-30.57, *P* = 0.0001) under a recessive model (Table 2), and similar associations were observed when the tumors were stratified by PR status (OR = 10.70, 95% CI 3.44-33.32, *P* < 0.0001). Notably, by assuming a recessive genetic model, this variant was associated with a markedly higher risk for the triple-negative subtype of breast cancer (OR = 18.65, 95% CI 5.42-64.13, *P* < 0.0001; Table 2) but not for the luminal-like or HER2+ subtypes. The disease features of the rs3734091-TT mutation carriers are presented in Supplemental Table 3. Homozygous rs3734091-TT tumors predominantly exhibited a triple-negative phenotype. Compared with patients with the rs3734091-GG (13%, 184 out of 1,444) or GT (12%, 21 out of 172) genotypes, there was a higher incidence of TNBC among patients harboring the rs3734091-TT genotype (50%, 8 out of 16) (Supplemental Figure 1). Together, these data implied that breast tumors of the rs3734091-TT genotype are more likely to have a triple-negative phenotype.

**Table 2:** Association of rs3734091 with breast cancer risk in the entire cohort by tumor molecular subtype

				Dominant model		Recessive model	
Study/subtypes	Genotype	Controls (%)	Cases (%)	OR^a^ (95% CI)	P^a^	OR^a^ (95% CI)	*P*^a^
**ER status**							
ER-negative							
	GG	1,421 (89.8%)	382 (86.4%)	1.40 (1.02-1.93)	0.044	9.43 (2.91-30.57)	**0.0001**
	GT	158 (10%)	50 (11.3%)				
	TT	4 (0.2%)	10 (2.3%)				
ER-positive							
	GG	1,421 (89.8%)	1,042 (89.1%)	1.08 (0.84-1.39)	0.54	2.40 (0.67-8.58)	0.17
	GT	158 (10%)	121 (10.3%)				
	TT	4 (0.2%)	6 (0.5%)				
**PR status**							
PR-negative							
	GG	1,421 (89.8%)	455 (86.2%)	1.43 (1.06-1.93)	0.021	10.70 (3.44-33.32)	**<0.0001**
	GT	158 (10%)	60 (11.4%)				
	TT	4 (0.2%)	13 (2.5%)				
PR-positive							
	GG	1,421 (89.8%)	969 (89.5%)	1.04 (0.81-1.35)	0.74	1.24 (0.28-5.61)	0.78
	GT	158 (10%)	111 (10.2%)				
	TT	4 (0.2%)	3 (0.3%)				
**HER2 (Neu) status**							
HER2-negative							
	GG	1,421 (89.8%)	1,104 (89.4%)	1.05 (0.82-1.35)	0.69	4.72 (1.52-14.65)	**0.0032**
	GT	158 (10%)	118 (9.6%)				
	TT	4 (0.2%)	13 (1.1%)				
HER2-positive							
	GG	1,421 (89.8%)	323 (85.2%)	1.55 (1.11-2.15)	0.012	3.21 (0.71-14.50)	0.15
	GT	158 (10%)	53 (14%)				
	TT	4 (0.2%)	3 (0.8%)				
**Luminal-like subtype**							
ER- and/or PR-positive							
	GG	1,421 (89.8%)	1,099 (89.4%)	1.05 (0.82-1.35)	0.69	2.30 (0.64-8.23)	0.19
	GT	158 (10%)	124 (10.1%)				
	TT	4 (0.2%)	6 (0.5%)				
**HER2-positive subtype**							
ER- and PR-negative and HER2-positive							
	GG	1,421 (89.8%)	164 (84.1%)	1.64 (1.08-2.50)	0.026	3.93 (0.71-21.78)	0.16
	GT	158 (10%)	29 (14.9%)				
	TT	4 (0.2%)	2 (1%)				
**TNBC subtype**							
ER-, PR-, and HER2-negative							
	GG	1,421 (89.8%)	184 (86.4%)	1.44 (0.94-2.21)	0.11	18.65 (5.42-64.13)	**<0.0001**
	GT	158 (10%)	21 (9.9%)				
	TT	4 (0.2%)	8 (3.8%)				

NOTE: Bold values denote *P* ≤ 0.01.

a The *P* value, OR, and 95% CI were calculated using logistic regression and were adjusted for age, age at menarche, age at primiparity, menopause status, family history of breast cancer, and BMI.

### Cells homozygous for p.Ala247Ser exhibit aberrant XRCC4 cytoplasmic localization

**Figure 1:**
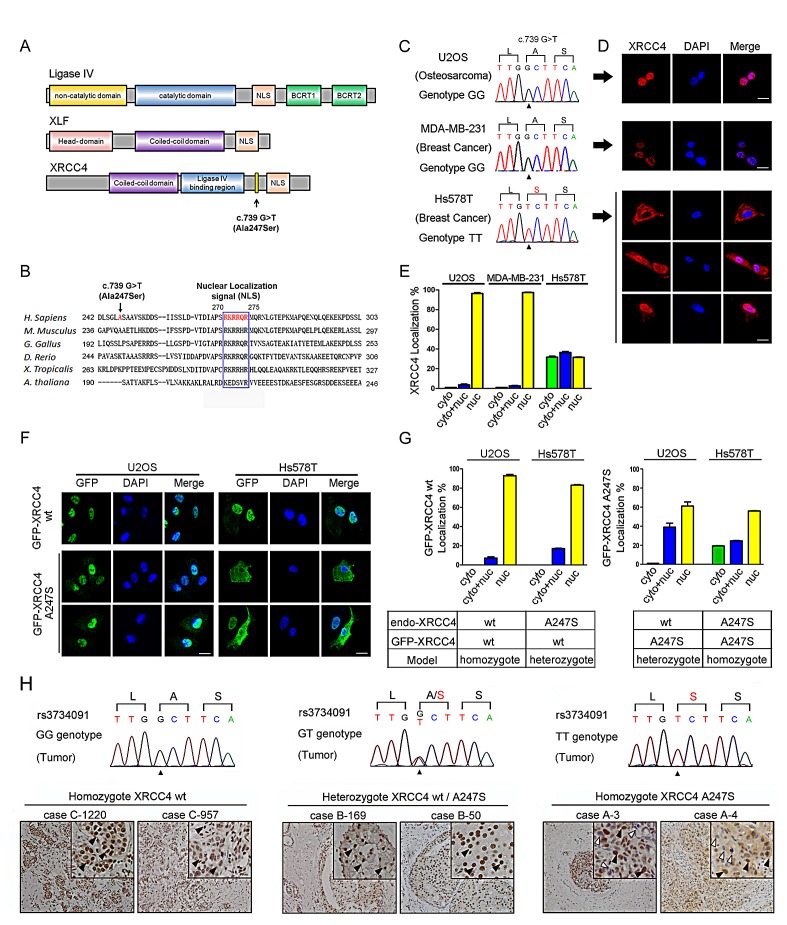
The homozygous *XRCC4* A247S mutation disrupts XRCC4 nuclear localization (A) Schematic diagram of the domain structures in XRCC4, XLF, and Ligase IV. NLS, nuclear localization signal; BRCT, BRCA1 C-terminal domain. Residue 247 (A247S) is located between the Ligase IV-binding region and the NLS in XRCC4. (B) Alignment of the conserved NLS region in *XRCC4* and the Ala247 codon (in red). (C) Chromatograms of the rs3734091 (c.739G>T) genotypes are displayed for different cell lines (U2OS, GG; MDA-MB-231, GG; Hs578T, TT). (D) Immunofluorescence of endogenous XRCC4 in U2OS (top), MDA-MB-231 (middle), and Hs578T (bottom) cells demonstrating the aberrant cytoplasmic localization of XRCC4^A247S^ in Hs578T cells. (E) Quantification of cells with a predominantly cytoplasmic, nuclear, or cytoplasmic/nuclear distribution of endogenous XRCC4, as shown in D. (F) Immunofluorescence of GFP-tagged XRCC4^wild-type (wt)^ and GFP-tagged XRCC4^A247S^ stably expressed in U2OS (left panel, endo-XRCC4^wt^) or Hs578T (right panel, endo-XRCC4^A247S^) cells, demonstrating that the cytoplasmic distribution of GFP-XRCC4^A247S^ only occurred in Hs578T cells in a homozygous model. (G) Quantification of cells with various GFP-tagged XRCC4 distribution patterns in homozygous and heterozygous models, as shown in F. The data are presented as the mean ± SEM. (H) The homozygous XRCC4^A247S^ mutant displays aberrant localization in breast cancer tissues. IHC analysis of endogenous XRCC4 in breast cancer tissue sections with different rs3734091 genotypes (cells positive for nuclear XRCC4, black arrow; cells negative for nuclear XRCC4, white arrow). Scale bar, 100 μm.

Because the rs3734091-TT genotype was associated with an increased risk of non-*BRCA1/2* breast cancer, we investigated the potential disease-inducing mechanism. The XRCC4-associated complex contains at least three components: XRCC4, XLF, and Ligase IV (Figure 1A). The T-allele of rs3734091 results in an exonic alteration (p.Ala247Ser, NM_003401.3: c.739 G>T) that is located between the Ligase IV-binding domain and the putative nuclear localization signal (NLS) (Figure 1A, top). In an alignment of the *XRCC4* sequences from different species, we noted that the Ala247Ser (A247S) mutation is adjacent to the conserved NLS (Figure 1B). To assess the potential influence of A247S on the NLS, we used immunofluorescence to examine the endogenous XRCC4 distribution patterns in three cell lines with different rs3734091 genotypes. Interestingly, among Hs578T cells harboring homozygous XRCC4^A247S^ protein (Figure 1C), there was population with aberrant cytoplasmic localization of endogenous XRCC4; conversely, the XRCC4^wild-type^ protein was completely localized to the nucleus in the homozygous U2OS and MDA-MB-231 cells (Figure 1C-E).

To further characterize the phenotype of the XRCC4^A247S^ protein, GFP-tagged XRCC4^wild-type^ or XRCC4^A247S^ was stably expressed in both U2OS and Hs578T cells (Figure 1F and G). Consistent with the endogenous XRCC4 distribution, GFP-tagged XRCC4^wild-type^ displayed a completely nuclear distribution in U2OS and Hs578T cells, whereas GFP-tagged XRCC4^A247S^ exhibited a partially cytoplasmic distribution in Hs578T cells. To our surprise, in an artificial heterozygous model (exogenous GFP-tagged XRCC4^A247S^ / endogenous XRCC4^wild-type^), GFP-tagged XRCC4^A247S^ was predominantly localized to the nucleus in U2OS cells. These findings suggested that heterozygous XRCC4^A247S^ was not sufficient to disrupt the nuclear localization of XRCC4; however, cells homozygous for XRCC4^A247S^ exclusively exhibited aberrant cytoplasmic localization.

To determine whether a similar mutator phenotype was present at the tissue level, we performed IHC to evaluate endogenous XRCC4 expression in human mammary tumors with different rs3734091 genotypes. We randomly selected 20 cases with the rs3734091-GG genotype, 20 cases with the GT genotype, and 6 cases with the TT genotype. Considering the potential effect of loss of heterozygosity (LOH) in these tissues, we extracted DNA from the selected tumors and sequenced the samples to identify the status of XRCC4 codons 246-248. Direct Sanger sequencing indicated that there was good concordance between the blood samples and breast tissues (Figure 1H). As expected, endogenous XRCC4 showed a uniform nuclear staining pattern in breast cancer tissues with the rs3734091-GG and GT genotypes, whereas various XRCC4 distribution patterns appeared in breast cancer tissue sections with the *XRCC4* rs3734091-TT genotype. Epithelial cells with negative nuclear staining were scattered among cells with positive nuclear staining in the breast cancer sections. These results further indicated that the homozygous XRCC4^A247S^ mutant displayed deficient nuclear XRCC4 localization.

### XRCC4^wild-type^ facilitates the nuclear localization of XRCC4^A247S^, compensating for the localization defect of XRCC4A^247S^ in heterozygous cells

**Figure 2:**
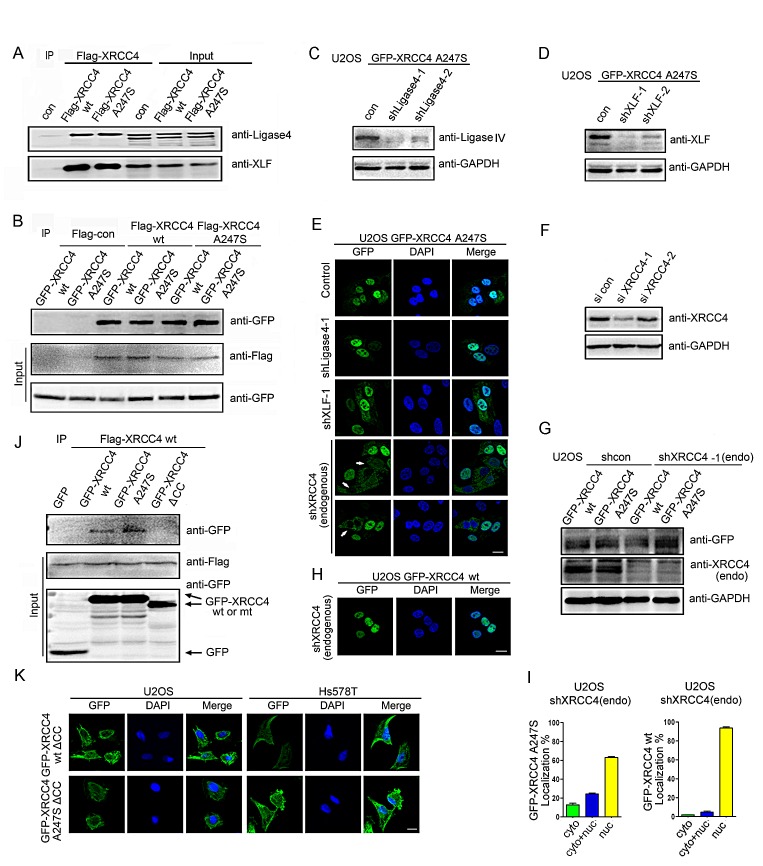
XRCC4^wild-type^ facilitates XRCC4^A247S^ nuclear localization (A) The A247S mutation did not impair the association between XRCC4 and XLF or Ligase IV. HEK-293T cells were transiently transfected with constructs containing FLAG-tagged XRCC4 wild-type (wt) or A247S. Immunoprecipitations were performed as indicated. (B) XRCC4^A247S^ interacted with XRCC4^wild-type^ and XRCC4^A247S^. 293T cells were simultaneously transiently transfected with FLAG-tagged or GFP-tagged XRCC4^wild-type^ or XRCC4^A247S^. Immunoprecipitations were performed as indicated. (C, D) U2OS cells stably expressing GFP-tagged XRCC4^A247S^ were exposed to lentivirus encoding shRNA control or shRNA targeting Ligase IV (C) or XLF (D). (E) U2OS cells stably expressing GFP-tagged XRCC4^A247S^ were exposed to lentivirus encoding control shRNA or shRNA targeting XLF, Ligase IV, or endogenous wild-type XRCC4. In the absence of endogenous XRCC4^wild-type^, GFP-tagged XRCC4^A247S^ displayed aberrant cytoplasmic localization (white arrows) in certain cells. (F) Identification of efficient RNAi sequences against the 3′-UTR of *XRCC4* mRNA. HEK-293T cells were transfected with control siRNA or XRCC4 siRNA-1 or siRNA-2, which targeted the 3′-UTR of *XRCC4* mRNA. Immunoblots for endogenous XRCC4 were performed as indicated. (G) U2OS cells stably expressing GFP-tagged XRCC4^A247S^ were treated with lentivirus encoding shRNA against endogenous wild-type XRCC4. Immunoblots were performed as indicated. (H) GFP-tagged XRCC4^wild-type^ displayed nuclear localization in the absence of endogenous XRCC4. (I) Quantification of cells with GFP-tagged XRCC4 expression as described in E (bottom) and H. The data are presented as the mean ± SEM. (J) The coiled-coil domain of XRCC4 was required for the autologous interactions. Lysates from HEK-293T cells expressing FLAG-tagged XRCC4^wild-type^ and GFP-tagged XRCC4^wild-type^, XRCC4^A247S^ or XRCC4^ΔCC^ were immunoprecipitated with a FLAG antibody. Immunoblotting was performed as indicated. (K) Immunofluorescence of U2OS and Hs578T cells stably expressing GFP-tagged XRCC4 mutants demonstrated that the coiled-coil domain of XRCC4 was essential for its nuclear localization regardless of the status of the A247S mutation.

The above findings prompted the question of why the homozygous XRCC4^A247S^ mutant caused abnormal localization whereas the heterozygous XRCC4^A247S^ mutant localized predominantly to the nucleus. We hypothesized that one or more components of the XRCC4-associated complex may facilitate XRCC4^A247S^ nuclear localization in heterozygous cells. Coimmunoprecipitation experiments demonstrated that XRCC4^A247S^ did not impair the interactions between XRCC4 and XLF or Ligase IV (Figure 2A). Additionally, XRCC4^A247S^ maintained the ability to form a dimer or polymer with XRCC4^wild-type^ or XRCC4^A247S^ (Figure 2B), suggesting that the A247S alteration did not affect the interactions between XRCC4 and its associated partners.

To identify which component in this complex facilitates XRCC4^A247S^ nuclear localization, we silenced Ligase IV and XLF in U2OS cells stably expressing GFP-tagged XRCC4^A247S^ (Figure 2C and D). Depleting Ligase IV and XLF did not affect the nuclear localization of GFP-tagged XRCC4^A247S^ (Figure 2E). Considering that XRCC4 forms a dimer or a polymer in the complex, we first identified an efficient siRNA sequence targeting the 3′-UTR of *XRCC4* mRNA (siXRCC4-1, Figure 2F). Next, we constructed lentivirus-based shRNA vectors harboring the siXRCC4-1 hairpin sequence and established stable cell lines expressing GFP-tagged XRCC4^A247S^; in this system, endogenous XRCC4 is silenced after lentivirus transduction (Figure 2G). Notably, in the absence of endogenous wild-type XRCC4, GFP-tagged XRCC4^A247S^ displayed an aberrant cytoplasmic expression pattern (Figure 2E and I). In contrast, the depletion of endogenous XRCC4 did not affect the nuclear localization of GFP-tagged XRCC4^wild-type^ (Figure 2H and I). These results indicated that endogenous XRCC4^wild-type^ facilitated the nuclear localization of XRCC4^A247S^, thus compensating for the localization defect in cells heterozygous for XRCC4^A247S^.

To further explore the importance of the autologous interactions of XRCC4, we introduced the GFP-tagged XRCC4^ΔCC^ mutant, which lacks the ability to bind to wild-type XRCC4 (Figure 2J), into U2OS and Hs578T cells. Interestingly, GFP-tagged XRCC4^ΔCC^ failed to localize to the nucleus in both cell lines (Figure 2K). Moreover, the XRCC4^ΔCC^ mutant with an exogenous SV40-NLS was not completely localized to the nucleus (Supplemental Figure 2), implying that the autologous interactions of XRCC4 are essential for its nuclear localization.

### The XRCC4^A247S^ variant impairs the DNA damage repair (DDR) pathway and genomic stability

**Figure 3:**
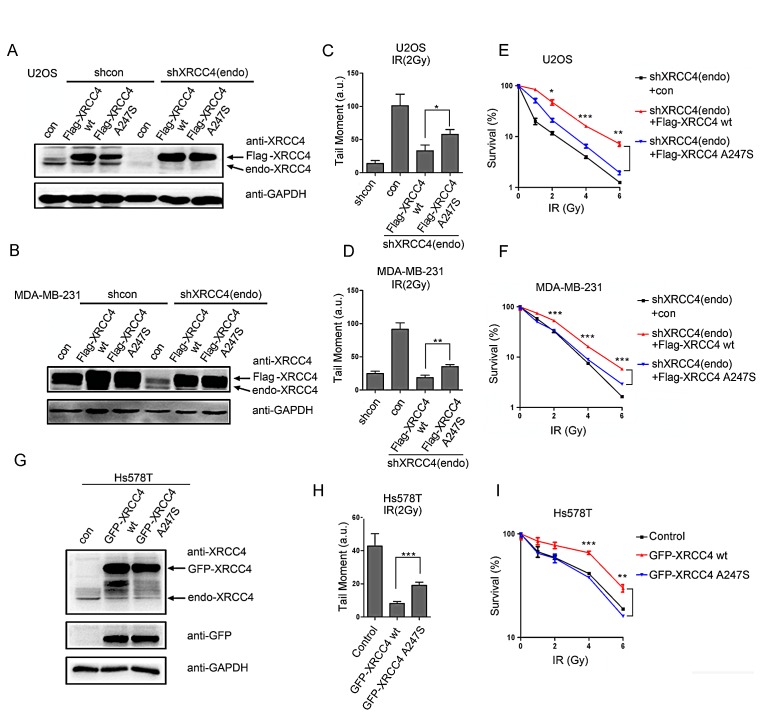
The XRCC4^A247S^ mutant impairs *XRCC4-*mediated DDR (A, B) Immunoblot analysis of XRCC4-depleted cells and cells rescued with shRNA-resistant FLAG-tagged XRCC4 (wild-type or A247S mutant) or controls (A, U2OS cells; B, MDA-MB-231 cells). Cells were sequentially infected with lentivirus expressing FLAG-tagged XRCC4 and shXRCC4-1. (C, D) XRCC4-depleted cells and XRCC4-rescued cells were treated with 2 Gy IR for a comet assay to quantify the DDR (C, U2OS cells; D, MDA-MB-231 cells). DNA damage was quantified by the tail moment. The data are presented as the mean ± SEM (*, *P* < 0.05; **, *P* < 0.01; a.u.: arbitrary units). (E, F) The various XRCC4-rescued cells were plated at a low density and irradiated with various doses of IR as indicated; colonies were counted after 14 days (E, U2OS cells; F, MDA-MB-231 cells). The data are presented as the mean ± SEM (*, *P* < 0.05; **, *P* < 0.01; ***, *P* < 0.001). (G) Hs578T cells were infected with lentivirus expressing GFP-tagged XRCC4 (wild-type or A247S mutant) or control. Immunoblotting experiments were performed using the indicated antibodies. (H) The comet assay demonstrated that GFP-tagged XRCC4^A247S^ could not compensate for the endogenous XRCC4 deficiency in Hs578T cells with regard to the DDR. The cells were irradiated (2 Gy) and subjected to a comet assay. The data are presented as the mean ± SEM (***, *P* < 0.001; a.u.: arbitrary units). (I) In response to IR, exogenous GFP-tagged XRCC4^A247S^ could not fully rescue XRCC4-deficient Hs578T cells compared with GFP-tagged XRCC4^wild-type^. The cells were plated at a low density and irradiated with various doses of IR as indicated; colonies were counted after 14 days. The data are presented as the mean ± SEM (**, *P* < 0.01; ***, *P* < 0.001).

XRCC4 is involved in the NHEJ pathway that participates in DSB repair and genomic maintenance [24]. Here, we developed XRCC4-depleted and derivative XRCC4-rescued cellular models in the U2OS and MDA-MB-231 cell lines (Figure 3A and B, as described in the Methods section). A comet assay was utilized to detect DSBs induced by ionizing radiation (IR) and to characterize cellular reversibility. As expected, XRCC4-depleted cells failed to efficiently repair IR-induced DSBs (Figure 3C and D and Supplemental Figure 3A). Notably, DSB repair in response to IR was fully restored in XRCC4^wild-type^-rescued cells but not in XRCC4^A247S^-rescued cells.

To further evaluate the impact of the A247S variant on DDR, we performed clonogenic assays on XRCC4-rescued cell lines. As shown in Figure 3E and F, XRCC4^wild-type^-rescued cells, but not XRCC4^A247S^-rescued cells, displayed improved cell survival after IR. In addition, XRCC4^wild-type^ expression in Hs578T cells reduced the sensitivity to IR compared with controls or cells expressing XRCC4^A247S^ (Figure 3G-I and Supplemental Figure 3B). These findings indicated that the A247S variant impaired the activity of XRCC4 within the DSB repair pathway.

**Figure 4:**
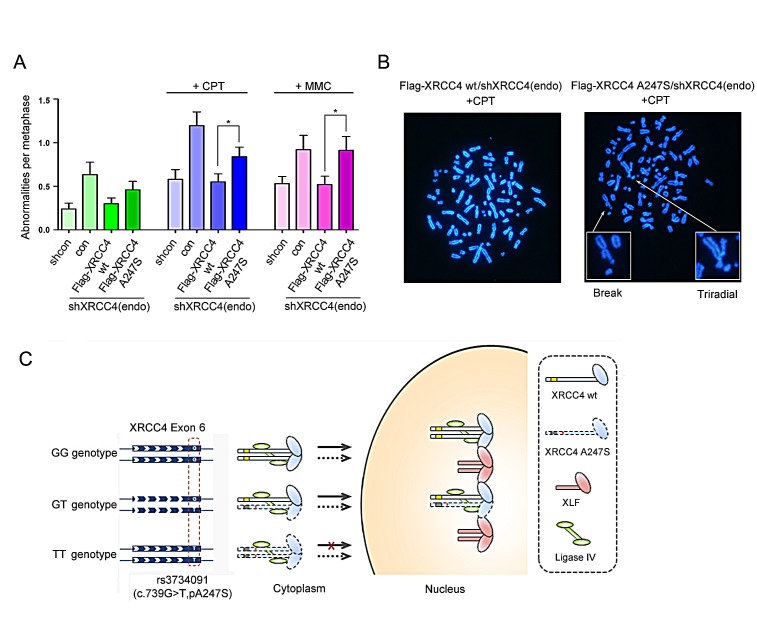
Genomic instability and sister chromatid exchange in cells expressing XRCC4^A247S^ (A) XRCC4-depleted U2OS cells expressing FLAG-tagged XRCC4^A247S^ had more chromosomal aberrations than cells expressing FLAG-tagged XRCC4^wild-type^. The number of chromosomal abnormalities per metaphase was scored for each group in a blinded manner. The data are presented as the mean ± SEM. (B) Representative images for A. (C) A model depicting the potential disease-promoting activity of the homozygous XRCC4^A247S^ mutant: under a genetic recessive model, the *XRCC4* germline variant rs3734091-TT partially disrupts XRCC4 nuclear localization and ultimately impairs the DDR pathway and decreases genome stability (see Discussion).

Considering the prominent role of the NHEJ pathway in maintaining genomic integrity [25], we investigated whether the A247S variant leads to chromosomal abnormalities and genomic instability. U2OS cells infected with control or shXRCC4 lentivirus were treated with MMC or CPT and then with nocodazole to synchronize the cells in the G2/M phase for a metaphase spread assay. As indicated in Figure 4A and B, XRCC4 depletion led to the accumulation of abnormal chromosomes in all three groups. Notably, wild-type XRCC4 expression reversed the accumulation of chromosomal abnormalities in XRCC4-depleted cells. In contrast, XRCC4^A247S^ expression did not completely compensate for the loss of endogenous XRCC4. The prevalence of chromosomal breaks and rearrangements was higher in XRCC4^A247S^-rescued cells than in XRCC4^wild-type^-rescued cells, suggesting that the A247S variant impaired DSB repair and reduced genomic integrity. Thus, the significantly increased chromosomal instability caused by the XRCC4^A247S^ variant provides biological evidence that XRCC4^A247S^ confers breast cancer susceptibility in homozygous carriers.

## DISCUSSION

To the best of our knowledge, this is the first study to investigate the associations between common variants of *XRCC4* and non-*BRCA1/2* breast cancer risk in eastern Chinese women. Consistent results from the two-stage genetic association study revealed that a recessive missense variant (rs3734091) of *XRCC4* was significantly associated with an increased risk of non-*BRCA1/2* breast cancer. Moreover, we discovered that the homozygous T allele of rs3734091 disrupted XRCC4 nuclear localization, elicited an aberrant DNA damage response, and ultimately resulted in genomic instability.

As illustrated in Figure 4C, our experimental results showed that *XRCC4* harboring the rs3734091-GG or GT genotypes fully localized to the nucleus and participated in DSB repair. However, replacing the GG or GT genotype with the TT genotype results in the homozygous A247S variant, which caused aberrant cytoplasmic localization of XRCC4 and deficient DSB repair, thus facilitating genomic instability. These data supported a disease-causing recessive model for how the *XRCC4* rs3734091-TT genotype, but not the GG or GT genotypes, is associated with an increased risk of breast cancer.

Exploring genetic risk factors related to breast cancer is important because identifying such factors might be useful for genetic counseling, risk prediction, and the development of preventive measures. Despite intense research, GWAS and next generation sequencing (NGS) exome studies have not identified additional major breast cancer susceptibility genes, such as *BRCA1* and *BRCA2* [1, 2, 26, 27]. It is likely that the remaining susceptibility is due to multiple low to moderate risk alleles of numerous genes that act in a polygenic model [28, 29]. The current study strongly suggested that the low and recessive population-specific genetic variant rs3734091 of *XRCC4* may influence the risk of non-*BRCA1/2* breast cancer, which could be missed by routine GWAS analyses of women of European ancestry. Interestingly, Chiu *et al.* [30] reported that there was no significant association between rs3734091 and breast cancer risk in a Taiwanese population; however, the small simple size and differences in the inclusion criteria may partially account for this discrepancy. Notably, the observed increment of breast cancer risk conferred by rs3734091 was independent because the sufficient sample size, multivariate adjustment for potential confounding factors, successful replication and functional demonstration minimized the type I errors. However, further analysis is necessary to validate our findings.

In our stepwise case-control study, rs3734091 was specifically associated with TNBC. Furthermore, tumors of *XRCC4* carriers, similar to BRCA1, were strongly associated with the triple-negative phenotype. A recent comprehensive analysis of The Cancer Genome Atlas (TCGA) program indicated that TNBC tumors show a high frequency of variants in homologous recombination (HR) DSB repair genes, including *TP53*, *BRCA1*, and *BRCA2* [31]. Deficiencies in other DDR pathways, such as base-excision repair (BER) [32] and nucleotide excision repair (NER) [33], have also been implicated in TNBC. Our results here provide additional evidence of the close relationship between defective NHEJ repair genes and the development of TNBC. Although the exact underlying mechanism remains to be elucidated, the allele frequencies of genetic variants with prominent function are likely to be low but could be associated with an increased cancer risk.

Genomic instability is one of the fundamental hallmarks of carcinogenesis [34]. XRCC4 is an essential modulator of the final ligation process in the NHEJ pathway of DSB repair in mammals [35]. XRCC4-knockout mice exhibit late embryonic lethality accompanied by defective lymphogenesis and neurogenesis [36]. In this study, cells homozygous for XRCC4^A247S^ exhibited a significant increase in chromosomal abnormalities, implying that the homozygous XRCC4^A247S^ status led to deficiencies in the DSB repair pathway, ultimately resulting in mammary carcinogenesis. Additionally, certain studies have reported that *XRCC4* rs3734091 is involved in the susceptibility to other malignant tumors besides breast cancer, such as hepatic carcinoma [17] and oral cancer [20]. Considering this fact, homozygous XRCC4^A247S^ might represent a general cause of mammalian cancers.

Although rs3734091 has been identified as a cancer-causing variant, it should be noted that the homozygous A247S variant does not fully abolish XRCC4 function. The mechanisms by which a portion of homozygous XRCC4^A247S^ maintains the capacity to be localized to the nucleus remain unknown. Ligase IV or XLF depletion did not disturb the nuclear localization of homozygous XRCC4^A247S^ (Supplemental Figure 4), implying that Ligase IV and XLF might not be involved in this process. One possibility is that the homozygous A247S alteration does not fully abolish the function of the NLS at the C-terminus of XRCC4. The C-terminal domain of XRCC4 has not been successfully crystallized due to its highly flexible structure [37, 38]. A recent study reported that SUMOylation of lysine 210 in XRCC4 regulates its nuclear localization and function in DSB repair [39]. Additionally, multiple serine residues at the C-terminus of XRCC4 have been identified as DNA-PKcs-dependent phosphorylation sites [40]. Therefore, it is possible that A247S alters the SUMOylation or phosphorylation of XRCC4 and thereby partially impairs the nuclear localization of XRCC4. More studies are warranted to elucidate the mechanism by which the A247S variant affects XRCC4 activity.

In conclusion, we combined epidemiologic and experimental studies to establish a role for *XRCC4* in the predisposition to non-*BRCA1/2* breast cancer in a Chinese population. Tumors with the homozygous rs3734091-TT genotype predominantly exhibited a triple-negative phenotype. We also demonstrated that homozygous XRCC4^A247S^ impaired DSB repair and decreased genomic integrity by disturbing XRCC4 nuclear localization. This work might implicate *XRCC4* as a novel susceptibility locus for breast cancer risk evaluation and prevention.

## MATERIALS AND METHODS

### Study populations and data collection for the two-step case-control study

All the participants in this study were genetically unrelated Han Chinese residing in Shanghai city and its surrounding areas. The first discovery case-control study included 562 patients with pathologically confirmed primary breast cancer who visited the Department of Breast Surgery at Fudan University Shanghai Cancer Center (FDSCC) between January 2005 and June 2008. During the same period, 504 controls who visited the Outpatient Department of FDSCC for breast cancer screening were matched to the breast cancer patients by age and region of residence. In an independent validation study, we collected an additional 1,202 patients via the same process between December 2008 and July 2010 and an additional 1,119 controls from a community-based breast cancer screening program, as described previously [41]. All the patients and controls with a family history of breast cancer were negative for *BRCA1/2* mutations, as we reported previously [5]. All of the controls were determined to be cancer-free after comprehensive examinations.

This study was approved by the Ethical Committee of the FDSCC, and each participant signed an informed consent document. After completing a written informed consent document, each participant donated approximately 5 ml of peripheral venous blood and was carefully interviewed to obtain epidemiological information. Supplemental Table 4 presents the detailed baseline characteristics of the enrolled subjects. There were no significant differences in the age and menopausal status distributions between the cases and controls in both data sets. However, the breast cancer patients were more likely to be younger at menarche and primiparity and to weigh more than the controls (*P* < 0.05). Moreover, a significant number of the breast cancer patients had a family history of first-degree relatives with breast cancer.

The clinicopathological diagnosis of breast cancer was determined by pathologists in the Department of Pathology. The estrogen receptor (ER), progesterone receptor (PR), and human epidermal growth factor receptor 2 (HER2) status was determined by immunohistochemistry (IHC). We defined the breast cancer IHC subtypes as the following: luminal-like (ER+ and/or PR+), HER2+ (HER2+, ER- and PR-), and triple-negative (ER-, PR-, and HER2-).

### Preliminary SNP screening and SNP selection

To obtain a general evaluation of the allele frequency of *XRCC4* SNPs in our study population, we performed a preliminary screening of genomic DNA from 20 randomly selected breast cancer patients and 20 unaffected controls and analyzed the coding sequence (CDS), promoter region (defined as the 2-kb sequence upstream of the transcriptional start site of *XRCC4*), and the 3′ untranslated region (3′ UTR) of *XRCC4* based on the NCBI genetic database. A total of 12 SNPs of *XRCC4* were found in this pilot screening (Supplemental Table 5). Among these 12 *XRCC4* SNPs, one SNP located at c.433C>G was novel; the other 11 SNPs had been previously reported in the NCBI dbSNP database (http://www.ncbi.nlm.nih.gov/). Using 5% as the cutoff criterion for the minor allele frequency (MAF) in Chinese subjects, four SNPs (rs3763063, rs1993947, rs16900150, and rs1805377) were excluded, and another four SNPs (rs2075685, rs2075686, c.433C>G, and rs1056503) were predicted as not potentially functional SNPs using SNP function prediction (FuncPred) software (http://snpinfo.niehs.nih.gov/snpfunc.htm). Finally, only two SNPs (rs56334522 and rs3734091) in the CDS and two SNPs (rs28360342 and rs2035990) in the 3′ UTR were selected for genotyping in the first study group.

### DNA preparation and genotyping

The genotyping for the four selected SNPs in the first set was performed using a Multiplex SNaPshot Kit (Applied Biosystems Inc., Foster City, CA, USA) according to standard protocols. The genotyping success rate was 96.4%, and the concordance rate was 100% for duplicate samples. This genotyping work was performed by Genesky Biotechnologies Inc. (Shanghai, China). In the validation set, the genotyping was performed using a TaqMan real-time PCR assay and a 7900 HT Sequence Detection System (Applied Biosystems Inc., USA) as described previously [42]. To ensure the accuracy of the genotyping, four positive controls (repeat samples) and four negative controls (without DNA template) were included in each of the 384-well plates. As a result, the genotyping rate was 98.1%, and the results of the repeated samples were 100% concordant.

### siRNA and shRNA constructs

siRNAs targeting the 3′-UTR of *XRCC4* mRNA and a non-targeting control siRNA were purchased from GenePharma Inc. (Shanghai, China). siRNA transfections were performed using Lipofectamine2000 (Invitrogen, Carlsbad, CA) according to the manufacturer's protocol. The siXRCC4-1 and siXRCC4-2 sequences were 5′ GCA GCC GCU AUU ACC GUA UTT 3′ and 5′ GAU GUU CAC UAG ACU AUG UTT 3′, respectively. The siXRCC4-1 sequence targeting the 3′-UTR of *XRCC4* mRNA was designed and cloned into a pLV construct to create the shXRCC4-1 lentivirus. shRNAs against XLF and Ligase IV were also designed and cloned into the pLV construct. The sequences of shXLF-1 and shXLF-2 were 5′ GCA GCC GCU AUU ACC GUA UTT 3′ and 5′ GAU GUU CAC UAG ACU AUG UTT 3′, respectively. The shLigase IV-1 and shLigase IV-2 sequences were 5′ GCA GCC GCU AUU ACC GUA UTT 3′ and 5′ GAU GUU CAC UAG ACU AUG UTT 3′, respectively. The shRNA constructs used for depleting XLF, Ligase IV, and endogenous XRCC4 were supplied by GeneChem Inc. (Shanghai, China).

### Expression constructs

The full-length and point mutants of human *XRCC4* were generated by PCR and subcloned into the pLenti6.2 vector (Invitrogen, Carlsbad, CA) carrying a FLAG epitope tag at the N terminus. The GFP-tagged expression constructs for wild-type and deletion/point mutants of *XRCC4* were established using a lentiviral vector harboring an N-terminal GFP tag, as previously described [43]. The *XRCC4* point mutants were generated with a Quick-Change II site-directed mutagenesis kit (Stratagene, La Jolla, CA) according to the manufacturer's protocol. *XRCC4* deletion mutants were generated by cloning the corresponding cDNA fragments into the above-mentioned vectors.

### Cell culture and stable cell lines

The human breast cancer cell lines (MDA-MB-231 and Hs578T), human bone osteosarcoma cell line (U2OS), and human kidney epithelial cell line (HEK-293T) were obtained from American Type Culture Collection (ATCC), and were maintained in complete growth medium as recommended by the distributor. Transient transfections were performed using Lipofectamine2000 (Invitrogen, Carlsbad, CA, USA). Stable cell lines over-expressing XRCC4 were generated by infecting cells with retrovirus or lentivirus containing various GFP-tagged or FLAG-tagged *XRCC4* sequences, followed by selection with puromycin or blasticidin. The XRCC4-deficient, XLF-deficient, and Ligase IV-deficient stable cell lines were generated by infecting cells with lentivirus containing shRNAs against the relevant sequences and then selecting the cells with puromycin. For XRCC4-depleted cells rescued with wild-type or mutants of XRCC4, the cells were first infected with viruses expressing wild-type or mutant XRCC4. Subsequently, the stable cell lines were infected with lentivirus containing the appropriate shRNAs targeting the 3′ UTR of *XRCC4*.

### Cell lysis and immunoprecipitation

Cells were lysed in NETN buffer (0.15 M NaCl, 1 mM EDTA, 50 mM Tris-HCl, pH 8.0, and 0.5% Nonidet P-40) containing protease and protein phosphatase inhibitors (1 mM NaF and 1 mM Na_3_VO_4_) as previously described [44]. FLAG immunoprecipitations were performed in the same buffer with FLAG (M2) beads (Sigma-Aldrich, St. Louis, MO) overnight at 4°C.

### Colony formation assay

This assay was performed as described previously [44]. Briefly, stable cell lines were seeded at a low density and irradiated with 1, 2, 4, or 6 Gy using a Cs^137^ radiation source. The cells were then incubated at 37°C for 14 days to allow for colony formation. The colonies were stained with 2% methylene blue and 50% ethanol, and colonies containing 50 or more cells were counted and analyzed using Student's t-test.

### Immunofluorescence

Cells grown on coverslips were fixed with 4% paraformaldehyde for 30 min at room temperature, permeabilized with 0.5% Triton X-100 for 5 min at 4°C, and incubated with primary antibodies for 2 h at 37°C. The slides were then incubated with Alexa 488-conjugated (green, Invitrogen) or Alexa 555-conjugated (red, Invitrogen) secondary antibodies. Images were captured with a confocal laser microscope (Leica TCS SP5 II). At least 100 cells were analyzed for each group. The experiments were performed in triplicate.

### Immunoblotting analysis and antibodies

Whole cell extracts were obtained using NETN lysis buffer. The proteins were separated by 10% SDS-PAGE and detected using the following primary antibodies according to the manufacturer's instructions: anti-XRCC4 (Abcam, MA), anti-XLF (Abcam, MA), anti-Ligase IV (Thermo Scientific), anti-FLAG (M2, Sigma-Aldrich), anti-GFP (Invitrogen), and anti-GAPDH (Proteintech).

### Immunohistochemistry

Paraffin breast tumor sections were subjected to immunohistochemical staining to ascertain the protein localization of XRCC4, XLF, and Ligase IV. The tumor sections were incubated with the following primary antibodies: anti-XRCC4 (1:500; Abcam, MA), anti-XLF (1:400; Abcam, MA), and anti-Ligase IV (1:600; Thermo Scientific). HRP-conjugated secondary antibodies were used, followed by colorimetric detection using an Envision detection kit (DAKO, Japan).

### Comet assay

Stable cell lines were seeded at 80% density and irradiated with 2 Gy using a Cs^137^ radiation source. After an overnight incubation, the cells were harvested for a comet assay using an OxiSelect™ Comet Assay Kit (Cell Biolabs Inc.). Comet images were visualized and captured at 200x magnification with a fluorescence microscope (Olympus IX51). The Olive Tail Moment (OTM) on each damaged cell was quantified using CASP 1.2.3 (CASP lab, Wroclaw). At least 50 cells were analyzed per sample.

### Metaphase spreads

A metaphase spread assay was performed as previously described [45]. Cells were incubated with 10 mM BrdU for 48 h after treatment with or without a DNA-damaging agent (2.5 nM camptothecin (CPT) or 20 nM mitomycin (MMC)). The cells were then treated with 0.15 μg/ml nocodazole for 3 h, lysed with 75 mM KCl, and fixed with a 3:1 methanol/acetic acid solution. Metaphases were dropped onto slides kept at 65°C, and then the slides were dried and stained with DAPI. The numbers of sister chromatid breaks, triradial chromosomes and quadriradial chromosomes per metaphase were counted. At least 50 metaphases were analyzed in each group.

### Statistical analyses

HWE was evaluated with χ^2^ tests for each SNP locus. The associations between alleles and breast cancer risk were determined using Pearson's χ^2^ test. Logistical regression was used to analyze the associations between genotype and breast cancer risk. The crude OR and the ORs adjusted for age, age at menarche, age of primiparity, menopausal status, family history of breast cancer and BMI were determined along with the 95% CIs. We also performed Student's t-tests to compare the continuous variables between two groups and ANOVA to compare the continuous variables among three or four groups. Statistical analyses were performed using SPSS (Statistical Package for the Social Sciences) version 12.0 (SPSS Inc., Chicago, IL, USA), SNPstats (Catalan Institute of Oncology, Catalonia, Spain), and GraphPad Prism 5 (GraphPad Software, Inc., La Jolla, CA). The linkage disequilibrium (LD) of the selected SNPs was constructed using the Haploview 4.2 program and is shown in Supplemental Figure 5.

Quanto (http://hydra.usc.edu/gxe) was used to estimate the statistical power of the present study. The MAF of rs3734091 (approximately 7% based on our genotyping results), the OR (3 or 3.5), the incidence of breast cancer in the study population (25 in 100,000 in Shanghai, China), and a recessive genetic model were used as the parameters. In the first test set, the sample size had a power of 36% and 47% to detect alleles with ORs of 3 and 3.5, respectively. In the validation set, the sample size had a power of 66% and 80% to detect alleles with ORs of 3 and 3.5, respectively. When combining the two case-control studies, the sample size had powers of 82% and 92% to detect alleles with ORs of 3 and 3.5, respectively.

### Funding

The work was supported by grants from the National Natural Science Foundation of China (81201531), the 2012 Shanghai Committee of Science and Technology Funds (12ZR1406200 and 12DZ2260100), the Shanghai Committee of Science and Technology Fund for 2013 Qimingxing Project (11QA1401400 to X.H.). The funders had no role in study design, data collection and analysis, decision to publish, or preparation of the manuscript.

### Competing Interests

The authors have declared that no competing interests exist.

## SUPPLEMENTARY MATERIAL, FIGURES AND TABLES


