# Supplementary material for: A recessive variant of XRCC4 predisposes to non-BRCA1/2 breast cancer in chinese women and impairs the DNA damage response via dysregulated nuclear localization
Source: Oncotarget. 2014 Oct 22;5(23):12218–32. doi: 10.18632/oncotarget.2623 (PMC4322983; doi:10.18632/oncotarget.2623)
Supplement: Supplementary file 1 [file oncotarget-05-12218-s001.pdf]

A recessive variant of *XRCC4* predisposes to non-B*RCA1/2* breast cancer in chinese women and impairs the DNA damage response via dysregulated nuclear localization

Supplementary Material

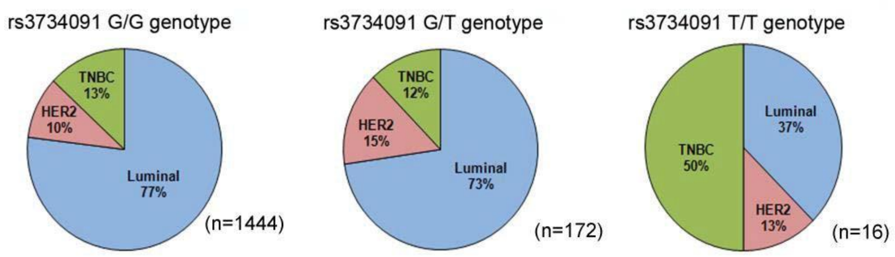

Supplemental Figure 1: Distribution of breast cancer molecular subtypes in various rs3734091 genotypes. The criteria for determining the molecular subtypes of breast cancer are described in the Materials and Methods section.

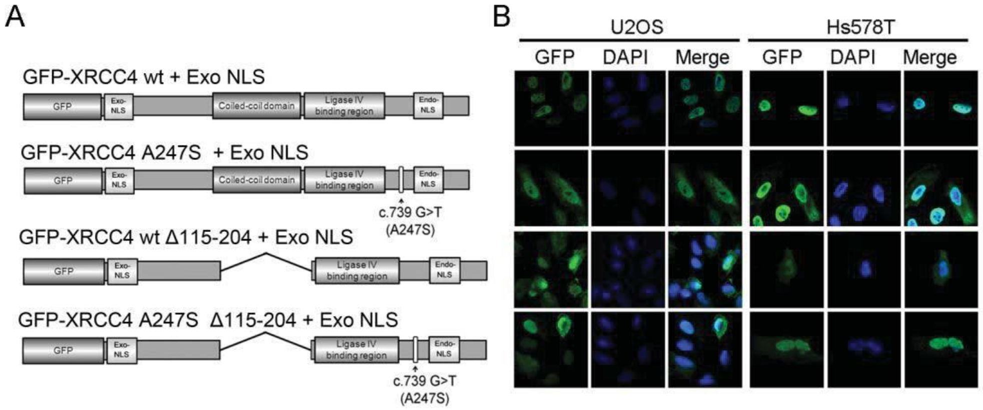

Supplemental Figure 2: An exogenous NLS protein sequence cannot fully compensate for the loss of the coiled-coil domain in XRCC4 nuclear localization. (**A**) A diagram of GFP-tagged wild-type XRCC4 and various mutant proteins with an exogenous NLS sequence. (**B**) Immunofluorescence of GFP-tagged wild-type XRCC4 and mutant proteins with exogenous NLS sequences was performed in U2OS and Hs578T cells.

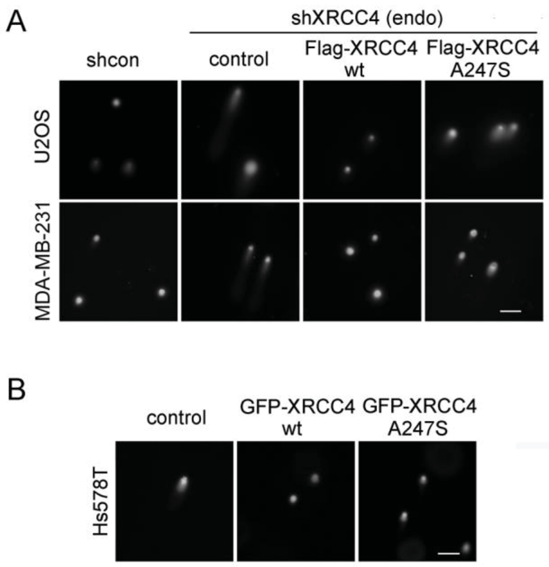

Supplemental Figure 3: Exogenous XRCC4^{A247S} fails to compensate for the depletion of endogenous XRCC4 $^{wild\text{-}type}$ in response to IR-induced DNA damage. (**A**) XRCC4^{A247S}-rescued cells and their respective wild-type counterparts were irradiated (2 Gy) and subjected to a comet assay (U2OS, top; MDA-MB-231, bottom). Representative electrophoresis images from three independent experiments are shown. Scale bar, 50 μm. (**B**) Representative comet assay images of XRCC4^{A247S}-rescued cells and wild-type Hs578T cells after 2 Gy of irradiation.

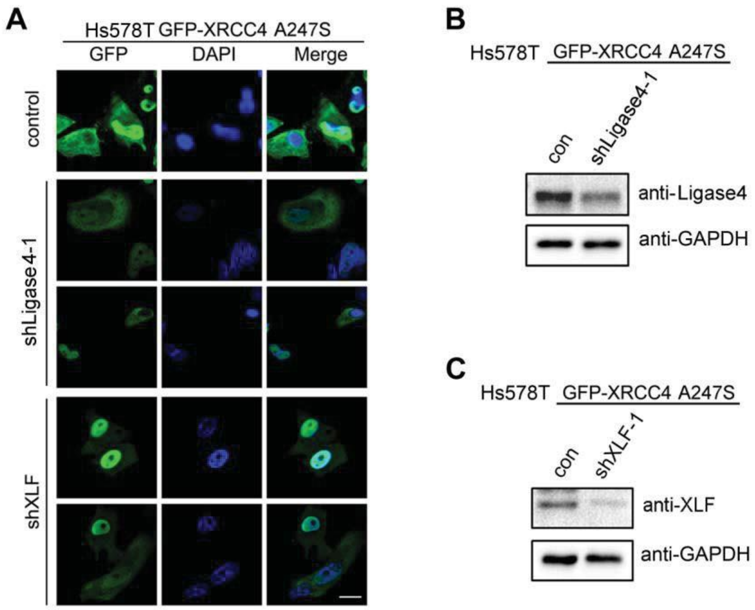

Supplemental Figure 4: The presence of XLF and Ligase IV is not required for the partial nuclear localization of XRCC4^{A247S}. (**A**) Hs578T cells stably expressing GFP-tagged XRCC4^{A247S} were treated with lentiviruses containing control shRNA and shRNA targeting XLF and Ligase IV. In a homozygous XRCC4^{A247S} model, the GFP-tagged XRCC4^{A247S} mutant did not change its localization in the absence of XLF or Ligase IV. (**B, C**) Immunoblotting was performed to evaluate the expression levels of XLF and Ligase IV in **A**.

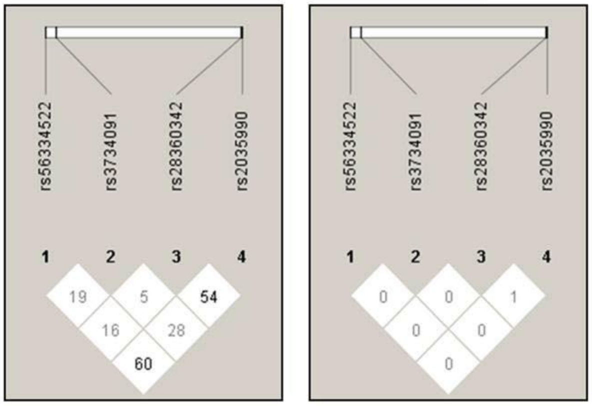

Supplemental Figure 5: LD analysis of the genetic variants in *XRCC4*. The top horizontal bar indicates the genetic region of the tested SNPs in *XRCC4*. The left triangle shows the LD calculated using the D’ measure; the right triangle shows the LD calculated using the r^2 measure. The value within each diamond represents the pairwise correlation between polymorphisms defined by the upper left and upper right sides of the diamond.

Supplemental Table 1: Allele frequencies of genotyped SNPs in *XRCC4*.

rs number	Gene	Location	Position	Protein Change	Minor Allele	Reference Allele	MAF		
							Cases (n[a],%)	Controls (n[a],%)	P^b
rs56334522	XRCC4	Exon 4	c.411	Asn137Lys	G	T	525 (<1.0%)	499(<1.0%)	1
rs3734091	XRCC4	Exon 6	c.739	Ala247Ser	T	G	543 (7%)	499 (6.3%)	0.778
rs28360342	XRCC4	3'UTR	c.*101	-	C	T	530 (3.2%)	483(3.8%)	0.434
rs2035990	XRCC4	3'UTR	c.*406	-	C	T	530 (50.3%)	498 (48.7%)	0.463

NOTE: [a] Number of allele

[b] *P* values are caculated by two-sided χ^2 test.

Abbreviations: UTR, untranslated region; MAF, minor allele frequency.

Supplemental Table 2: Associations between selected SNPs genotypes in *XRCC4* and breast cancer risk.

SNP	Genotype	Case(n)	Control(n)	OR[a] (95% CI)	OR[b] (95% CI)	Dominant model OR[c] (95% CI)	P^c	Recessive model OR[c] (95% CI)	P^c
rs56334522	TT	523	495	reference	reference	reference	NA	reference	NA
	TG	5	4	1.18 (0.32-4.43)	1.09 (0.29-4.13)	NA		NA	
	GG	0	0	NA	NA				
rs3734091	GG	475	435	reference	reference	reference	0.99	reference	**0.019**
	GT	64	63	0.93 (0.64-1.36)	0.93 (0.64-1.36)	1.00 (0.69-1.45)		NA (0.00-NA)	
	TT	4	0	NA (0.00-NA)	NA (0.00-NA)				
rs28360342	TT	496	447	reference	reference	reference	NA	reference	NA
	TC	34	36	0.85 (0.52-1.38)	0.89 (0.54-1.45)	NA		N/A	
	CC	0	0	NA	NA				
rs2035990	TT	136	140	reference	reference	reference	0.44	reference	0.7
	TC	256	233	1.13 (0.84-1.52)	1.11 (0.82-1.50)	1.12 (0.84-1.48)		1.06 (0.80-1.40)	
	CC	138	125	1.14 (0.81-1.59)	1.13 (0.80-1.59)				

NOTE: Bold values denote $P \leq 0.05$.

[a] Unadjusted odds ratio (OR) and 95% confidential interval (CI) calculated by logistic regression.

[b] Adjusted for age, age at menarche, menopause status, family history of breast cancer and BMI.

[c] *P* value, OR and 95% CI calculated by logistic regression and adjusted for age, age at menarche, age at primiparity, menopause status, family history of breast cancer and BMI.

Major genotype is indicated as reference.

Abbreviations: NA, not applicable.

Supplemental Table 3: Presentation of disease characteristics in patients with rs3734091 TT genotype.

Case No.	Patient ID	Morphology	Receptor Status	TNM	Age at diagnosis (years)	Family history of breast cancer and/or ovarian cancer	Molecular Subtypes
A-1	22**43	IDC	ER-,PR-,Her2-	T2N0M0	50	Negative	TNBC
A-2	22**90	IDC	ER-,PR-,Her2-	T2N1M0	40	Negative	TNBC
A-3	22**20	DCIS	ER-,PR-,Her2-	T2N0M0	54	Positive	TNBC
A-4	24**35	IDC	ER-,PR-,Her2-	T1N0M0	60	Positive	TNBC
A-5	25**70	IDC	ER-,PR-,Her2-	T2N0M0	49	Positive	TNBC
A-6	26**95	Adenocarcinoma	ER-,PR-,Her2-	T2N0M0	50	Negative	TNBC
A-7	27**66	IDC	ER-,PR-,Her2-	T2N1M0	55	Negative	TNBC
A-8	31**01	IDC	ER-,PR-,Her2-	T2N1M0	32	Negative	TNBC
A-9	26**49	IDC	ER-,PR-, Her2+	T2N0M0	43	Negative	Her2
A-10[a]	27**70	IDC	Right: ER-,PR-, Her2+<br>Left: ER-,PR-, Her2+	T2N2M0	53	Positive	Her2
A-11	21**06	IDC	ER+,PR-,Her2-	T1N0M0	51	Negative	Luminal
A-12	22**21	ILC	ER+,PR-,Her2-	T1N1M0	44	Positive	Luminal
A-13	22**15	IDC	ER+,PR+, Her2-	T1N1M0	45	Negative	Luminal
A-14	22**19	IDC	ER+,PR+,Her2-	T3N1M0	52	Negative	Luminal
A-15	25**21	IDC	ER+,PR-,Her2-	T1N1M0	61	Negative	Luminal
A-16	26**47	IDC	ER+,PR+,Her2+	T3N3M0	44	Negative	Luminal

Abbreviations: IDC, Invasive Ductal Carcinoma; DCIS, Ductal carcinoma in situ; ILC, Invasive lobular carcinoma; ER, estrogen receptor; PR, progesterone receptor; TNBC, triple-negative breast cancer.

TNM: Tumor-Node-Metastasis.

[a] Bilaterial Primary Breast Cancer.

SupplementalTable4: .Summarycharacteristicsoftheparticipants.

**Variables**	**Discoveryset,n(%)**		P^{a}	**Validationset,n(%)**		P^{a}
	**Patients (n=562)**	**Controls (n=504)**		**Patients (n=1202)**	**Controls (n=1119)**	
Age			0.941			0.534
≤50	369(65.7)	332(65.9%)		739(61.5%)	702(62.7%)	
>50	193(34.3%)	172(34.1%)		463(38.5%)	417(37.3%)	
Age at menarche			**0.004**			**0.001**
≤13	241(42.9%)	173(34.3%)		472(39.3%)	366(32.7%)	
>13	321(57.1%)	331(65.7%)		730(60.7%)	753(67.3%)	
BMI			**0.012**			**0.001**
≤24	304(54.1%)	311(61.7%)		646(53.7%)	675(60.3%)	
>24	258(45.9%)	193(38.3%)		556(46.3%)	444(39.7%)	
Age of primiparity			**0.013**			**0.007**
≤24	297(52.8%)	228(45.2%)		637(53.0%)	530(47.4%)	
>24	265(47.2%)	276(54.8%)		565(47.0%)	589(52.6%)	
Menopause			0.526			0.176
No	335(59.6%)	310(61.5%)		719(59.8%)	700(62.6%)	
Yes	227(40.4%)	194(38.5%)		483(40.2%)	419(37.4%)	
Family history			0.011			0.000
Yes	63(11.2%)	34(6.7%)		151(12.6%)	91(8.1%)	
No	499(88.8%)	470(93.3%)		1051(87.4%)	1028(91.9%)	

NOTE: Bold values denote $P \leq 0.05$.

[a] Two-sided χ^2 test for distributions between cases and controls.

Supplemental Table 5: Identification of the germlime variations in *XRCC4* gene region among 20 breast cancer patients and 20 healthy controls.

No.	dbSNP ID	Location	Nucleotide change	Position	MAF	Reported MAF in dbSNP
1	**rs3763063**	Promoter	c.-2039A>G	Untranlated 5'UTR	0.045	0.37
2	**rs1993947**	Promoter	c.-1582G>C	Untranlated 5'UTR	0.042	0.073
3	**rs16900150**	Promoter	c.-887G>A	Untranlated 5'UTR	0.046	0.017
4	**rs2075685**	Promoter	c.-827G>T	Untranlated 5'UTR	0.264	0.385
5	**rs2075686**	Promoter	c.-746C>T	Untranlated 5'UTR	0.3	0.093
6	**rs56334522**	Exon 4	c.411T>G	N137K	0.006	0.005
7	-	Exon 4	c.433C>G	Q145E	0.015	ND
8	**rs3734091**	Exon 6	c.739G>T	A247S	0.077	0.037
9	**rs1805377**	Intron 7	c.894-7G>A	Intronic	0.046	0.394
10	**rs1056503**	Exon 7	c.921T>G	S307S	0.136	0.394
11	**rs28360342**	Exon 7	c.*101T>C	Untranlated 3'UTR	0.057	0.013
12	**rs2035990**	Exon 8	c.*406T>C	Untranlated 3'UTR	0.449	0.203

NOTE: The variations found in this study are named according to the XRCC4's reference sequence NM_022550.2.

Abbreviations: MAF minor allele frequency; ND not detected.
